# Expression and Functional Characterization of Smyd1a in Myofibril Organization of Skeletal Muscles

**DOI:** 10.1371/journal.pone.0086808

**Published:** 2014-01-23

**Authors:** Jie Gao, Junling Li, Bao-Jun Li, Ezra Yagil, Jianshe Zhang, Shao Jun Du

**Affiliations:** 1 Institute of Marine and Environmental Technology, Department of Biochemistry and Molecular Biology, University of Maryland School of Medicine, Baltimore, Maryland, United States of America; 2 School of Chinese Materia Medica, Guangzhou University of Chinese Medicine, Guangdong, China; 3 Department of Biochemistry, Tel-Aviv University, Tel-Aviv, Israel; 4 Department of Bioengeneering and Environmental Science, Changsha University, Hunan, China; Institute of Molecular and Cell Biology, Singapore

## Abstract

**Background:**

Smyd1, the founding member of the Smyd family including Smyd-1, 2, 3, 4 and 5, is a SET and MYND domain containing protein that plays a key role in myofibril assembly in skeletal and cardiac muscles. Bioinformatic analysis revealed that zebrafish genome contains two highly related *smyd1* genes, *smyd1a* and *smyd1b*. Although Smyd1b function is well characterized in skeletal and cardiac muscles, the function of Smyd1a is, however, unknown.

**Methodology/Principal Findings:**

To investigate the function of Smyd1a in muscle development, we isolated *smyd1a* from zebrafish, and characterized its expression and function during muscle development via gene knockdown and transgenic expression approaches. The results showed that *smyd1a* was strongly expressed in skeletal muscles of zebrafish embryos. Functional analysis revealed that knockdown of *smyd1a* alone had no significant effect on myofibril assembly in zebrafish skeletal muscles. However, knockdown of *smyd1a* and *smyd1b* together resulted in a complete disruption of myofibril organization in skeletal muscles, a phenotype stronger than knockdown of *smyd1a* or *smyd1b* alone. Moreover, ectopic expression of zebrafish *smyd1a* or mouse *Smyd1* transgene could rescue the myofibril defects from the *smyd1b* knockdown in zebrafish embryos.

**Conclusion/Significance:**

Collectively, these data indicate that Smyd1a and Smyd1b share similar biological activity in myofibril assembly in zebrafish embryos. However, Smyd1b appears to play a major role in this process.

## Introduction

Members of the Smyd family are newly identified proteins that have been implicated in diverse biological functions in embryonic development and cancer [1]. Currently, five *smyd* genes (Smyd1, −2, −3, −4, and −5) have been identified in vertebrates based on the presence of both SET and MYND domains in their protein sequences [1]. Smyd1, also known as skm-Bop, represents the first identified member of the Smyd family [2], [3]. Smyd1 is specifically expressed in skeletal and cardiac muscles and plays a key role in muscle development and embryonic survival in mice and zebrafish [4]–[6]. Targeted disruption of the *smyd1* gene resulted in defective cardiac morphogenesis and early embryonic lethality of mouse embryos [4]. Knockdown or mutation of *smyd1b* gene in zebrafish led to disruption of myofibril organization in skeletal and cardiac muscles in zebrafish embryos [5], [6].

The *smyd1*gene is a direct downstream gene target of myogenic regulatory factors MyoD, Myogenin and Mef2 that control the muscle specific expression of *smyd1* in skeletal muscles during embryogenesis and in adult muscle tissues [7]–[10]. A recent report showed that *smyd1* expression is also regulated by serum response factor (SRF) through direct binding to the promoter region of *smyd1* [11]. In addition, *smyd1* gene expression can be repressed by Hepatoma-derived growth factor through interaction with a transcriptional co-repressor C-terminal binding protein (CtBP) [12]. Consistent with the idea of being a downstream factor of MyoD and Mef2, loss of Smyd1 function had no effect on *myoD* and *myogenin* gene expression and myoblast specification [5]. However, loss of Smyd1 function resulted in defective sarcomere organization in myofibers of skeletal and cardiac muscles, suggesting that Smyd1 is required in the late stage of muscle cell differentiation and myofiber maturation [5], [6].

At present, little is known about the mechanism by which Smyd1 functions in myofibrillogenesis. *In vitro* studies have shown that Smyd1 has a histone methyltransferase (HMTase) activity [5], [13], and could function as a transcriptional repressor in a histone deacetylase (HDAC)-dependent manor [4], [14]. However, Just and colleagues reported recently that the Smyd1 mutant lacking the HMTase activity was biologically active in myofibril assembly [6], arguing against Smyd1 being a HMTase activity dependent transcriptional regulator. Interestingly, Just and colleagues showed that GST-tagged Smyd1 was capable of pulling down skeletal muscle-specific myosin heavy chain [6]. Consistent with a potential role of Smyd1 outside of the nucleus, a nuclear to cytoplasmic translocation was observed during myoblast differentiation into myotubes [15], and Smyd1 is localized on the M-lines of sarcomeres although the biological significance of the sarcomeric localization is not clear [6], [16].

Recent studies demonstrated that zebrafish genome contains two highly related *smyd1* genes, *smyd1a* and *smyd1b* [17]. Most of the previous studies were focused on *smyd1b*, very little is known about *smyd1a*. It is not clear whether Smyd1a plays a similar role as Smyd1b in myofibrillogenesis. To investigate the function of Smyd1a in myofibril assembly, we isolated *smyd1a* from zebrafish, and characterized its expression and function during muscle development. The results showed that *smyd1a* was specifically expressed in skeletal muscles of zebrafish embryos. *smyd1a* expression came several hours later than *smyd1b* during myogenesis in zebrafish embryos. Functional analysis revealed that knockdown of *smyd1a* alone had little effect on myofibril assembly in zebrafish skeletal muscles. However, knockdown of *smyd1a* and *smyd1b* together resulted in a stronger phenotype in myofibril disorganization. Moreover, the myofibril defects from *smyd1b* knockdown could be rescued by an ectopic expression of the zebrafish *smyd1a* or mouse *Smyd1* transgene. Together, these data indicate that Smyd1a and Smyd1b share similar biological activity in myofibril assembly although the function of Smyd1b appears to be more critical.

## Results

### 1. Characterization of Smyd1a in Zebrafish

Sequence analysis revealed that zebrafish genome contains two highly related *smyd1* genes (*smyd1a* and *smyd1b*) with similar gene structures [17]. *smyd1a* and *smyd1b* are believed to be generated by gene duplication. The zebrafish *smyd1a* is located on chromosome 5, whereas *smyd1b* is located on chromosome 8 (Figure S1). Sequence analysis revealed a strong synteny arrangement of zebrafish *smyd1a* gene and human *smyd1* gene with the *thronine synthase like 2* gene (THNSL2) and *fatty acid binding protein1* gene (FABP1) in zebrafish and mouse genome (Figure S1). A similar synteny arrangement was found with the zebrafish *smyd1b* and *fatty acid binding protein 1b* like gene (*fabp1b*). However, no synteny arrangement was found with the *smyd1b* gene and *thronine synthase like* 2 gene on chromosome 8 in zebrafish. Sequence alignment revealed that zebrafish Smyd1a contains the highly conserved SET and MYND domains involved in protein methylation and protein-protein interactions, respectively (Figure S1). Both Smyd1a and Smyd1b share high sequence identity with Smyd1 from other vertebrate species (Figure S2), although only *smyd1* gene has been identified in mice and humans.

### 2. Temporal and Spatial Expression of Smyd1a in Zebrafish Embryos

**Figure 1:**
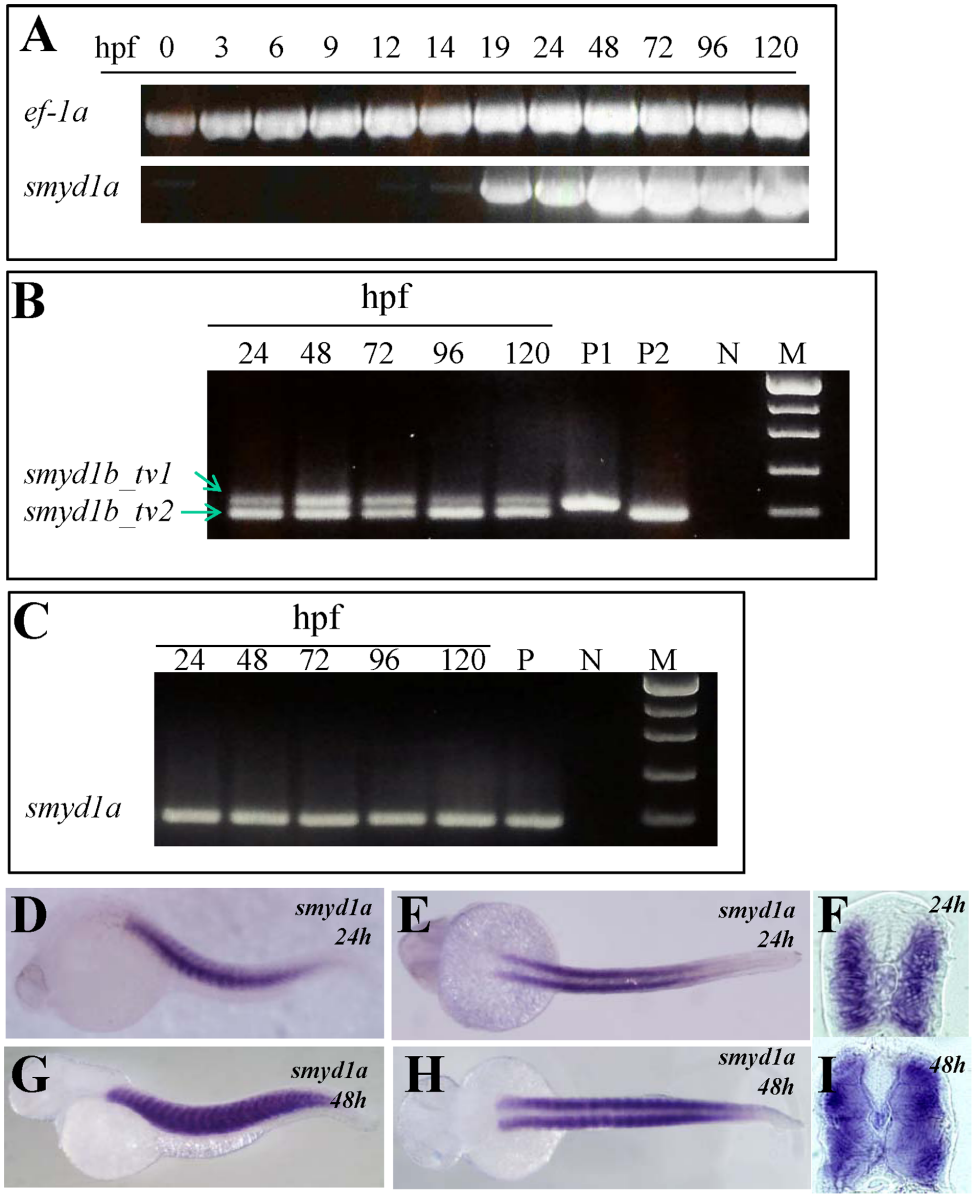
The temporal and spatial pattern of *smyd1a* expression in zebrafish embryos. **A.** RT-PCR results show temporal expression of *smyd1a* in zebrafish embryos from fertilization to day 5. *Elongation factor 1-alpha (ef-1α)* was used as control. **B.** RT-PCR analysis shows the alternative splicing of *smyd1b* exon 5 generating two isoforms of *smyd1b*, *smyd1b*_tv1 and *smyd1b*_tv2. **C.** RT-PCR analysis shows the lack of alternative splicing of exon 5 in *smyd1a* in zebrafish embryos. **D–I.** Whole mount in situ hybridization shows the spatial pattern of *smyd1a* mRNA expression using a dig-labeled antisense probe. *smyd1a* expression was detected in skeletal muscles of zebrafish embryos at 24 (D, E, F) and 48 (G, H. I) hpf. D, G represent the side view; E, H repre4sent the dorsal view; F, I represent the cross sections.

It has been reported previously that both *smyd1a* and *smyd1b* are specifically expressed in developing muscles of zebrafish embryos [5], [9], [17]. To compare their patterns of expression during embryonic development, we carried out a temporal and spatial expression analysis by RT-PCR and whole mount in situ hybridization in zebrafish embryos. Data from the expression analysis showed that *smyd1a* and *smyd1b* had different pattern of temporal expression. *smyd1a* was expressed several hours later than *smyd1b* in zebrafish embryos. *smyd1b* expression was first detected at 6 hours post-fertilization (hpf) with a strong expression starting around 9 hpf [5]. In contrast, *smyd1a* expression could not be detected until 19 hpf (Fig. 1A).

Previous studies have demonstrated that *smyd1b* encodes two muscle-specific isoforms of mRNA transcripts, designated as *smyd1b*_tv1 and *smyd1b*_tv2 [5], [16]. *smyd1b*_tv1 and *smyd1b*_tv2 are generated by alternative splicing of the 39 bp exon 5. *smyd1b*_tv1 transcript contains the 39 bp sequence from exon 5, whereas *smyd1b*_tv2 transcript lacks this 39 bp sequence and thus is 13 aa shorter than *smyd1b*_tv1 [5], [16]. Sequence analysis revealed that the zebrafish *smyd1a* gene also contained the small exon 5 of 39 bp. To test whether exon 5 could be alternatively spliced in *smyd1a*, we performed RT-PCR analysis using cDNA from zebrafish larvae of different stages. In contrast to *smyd1b* where two different isoforms were amplified (Fig. 1B), only the longer isoform of *smyd1a* was found to be expressed (Fig. 1C), suggesting that exon 5 in *smyd1a* was not alternatively spliced in zebrafish embryos.

To determine whether *smyd1a* expression is restricted to muscle cells, we analyzed its spatial pattern of expression by whole mount in situ hybridization. The data showed that similar to *smyd1b*, *smyd1a* is specifically expressed in skeletal muscles of zebrafish embryos (Fig. 1D–I). However, unlike *smyd1b,* little or no expression was detected for *smyd1a* transcripts in cardiac muscles (Fig. 1D, G).

### 3. Knockdown of *smyd1a* Expression in Zebrafish Embryos had Little Effect on Muscle Development

**Figure 2:**
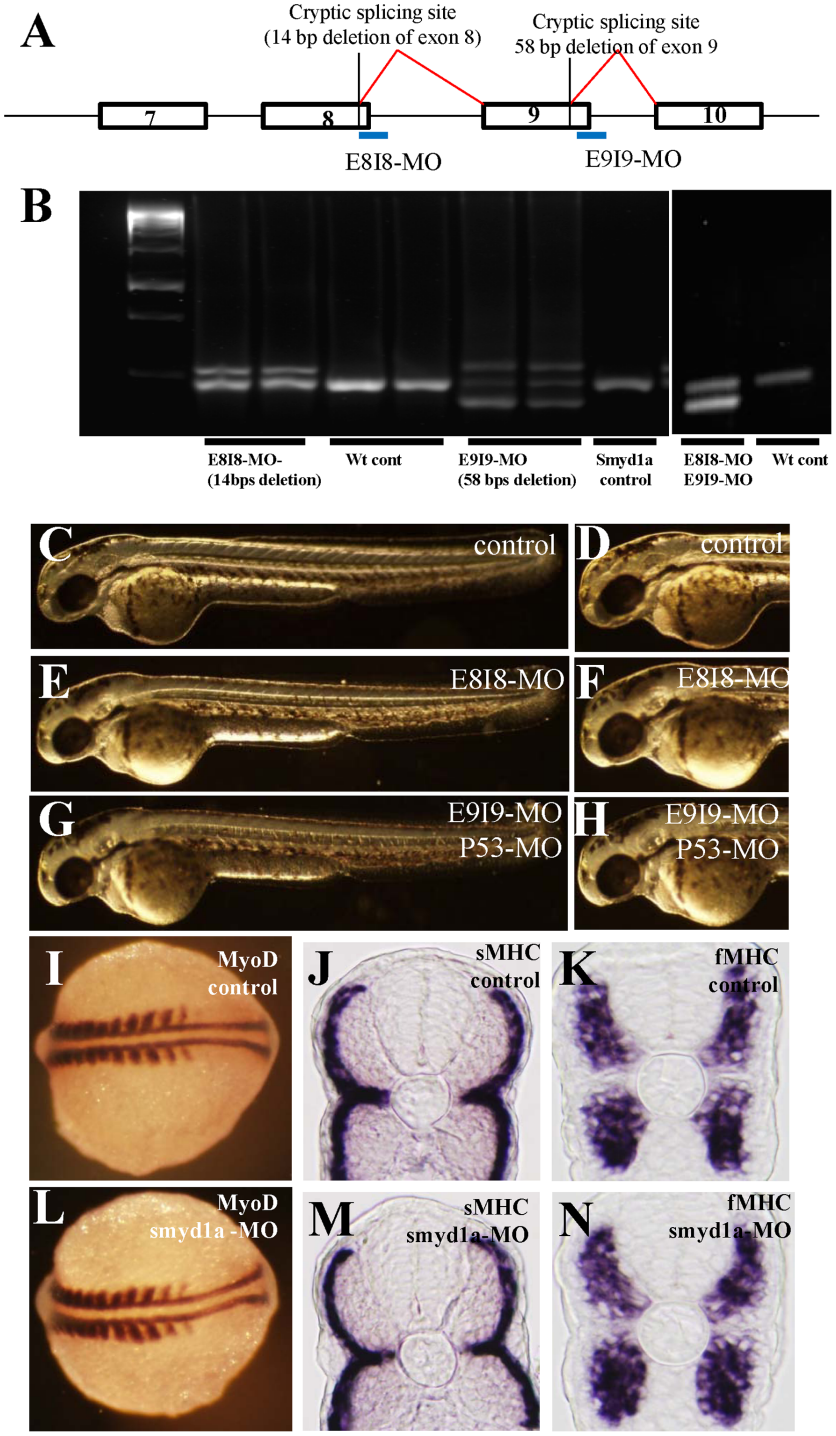
Knockdown of *smyd1a* splicing by E8I8-MO and E9I9-MO. **A.** Location of the E8I8-MO and E9I9-MO splicing blockers at the junction of exon8/intron8 and exon 9/intron 9, respectively. Defective splicing using a cryptic splicing site in exon 8 in E8I8-MO injected embryos resulted in a deletion of 14. Defective splicing using a cryptic splicing site in exon 9 in E9I9-MO injected embryos resulted in a deletion of 58 bp. **B.** RT-PCR showing the defective splicing induced by the E8I8-MO or E9I9-MO or both MOs. Compared with the PCR results from the wild type (wt) control embryos where a single band was generated, two bands were observed in the E8I8-MO injected embryos. Sequence analysis revealed that the smaller band contained a 14 bp deletion at the end of exon 8. The upper band represented the heteroduplex formed by the normal and defectively spliced products. Similarly, three bands were detected in E9I9-MO injected embryos. Sequence analysis revealed that the smaller band contained a 58 bp deletion at the end of exon 9. The middle band was the normal spliced product, whereas the upper band represented the heteroduplex formed by the normal and defectively spliced products. Two major bands were detected in E8I8-MO and E9I9-MO co-injected embryos. Sequence analysis revealed that the smaller band resulted from defective splicing. **C–H.** Morphology of *smyd1a* knockdown embryos at 48 hpf. Zebrafish embryos injected with control MO (C, D), E8I8-MO (E, F) or E9I9-MO (G, H) at 48 hpf. **I–N.** Knockdown of *smyd1a* expression had no effect on myoblast specification and early differentiation of slow and fast myofibers. *In situ* hybridization showing normal MyoD expression in control-MO (I) or *smyd1a* MO (L) injected embryos at 14 hpf. Cross views of slow (J, M) or fast (K, N) MHC expression in control (J, K) or *smyd1a* MO (M, N) injected embryos at 24 hpf.

To determine whether *smyd1a* plays a role in muscle development, we knocked down *smyd1a* expression in zebrafish embryos using two *smyd1a*-specific antisense morpholino oligos, E8I8-MO and E9I9-MO. The E8I8-MO and E9I9-MO were specifically targeted to the sequences at the exon-8/intron-8, or exon-9/intron-9 junctions, respectively (Fig. 2A). RT-PCR analysis confirmed that both E8I8-MO and E9I9-MO could knock down the normal splicing of *smyd1a* transcripts and resulted in the production of defectively spliced smyd1a mRNA (Fig. 2B). However, the knockdown was not complete with an efficiency of approximately 50% (Fig. 2B). Sequence analyses revealed that E8I8-MO caused a defective splicing at the exon 8 and intron 8 junction, resulting in a 14 bp deletion at the end of exon 8 (Fig. 2A). This 14 deletion caused a reading frame shift, leading to the production of a mutant protein without the 105 aa C-terminal sequence. Similarly, the E9I9-MO caused a defective splicing at the end of exon 9 with a 58 bp deletion (Fig. 2A), which resulted in the production of a mutant protein without the last 63 aa C-terminal sequence.

The *smyd1a* knockdown embryos were examined morphologically for several days following the MO injection. Unlike the *smyd1b* knockdown embryos that had no skeletal and cardiac muscle contraction, the *smyd1a* knockdown embryos had normal skeletal and cardiac muscle contraction when observed at 24, 48 and 72 hpf. The E8I8-MO injected embryos appeared morphologically normal (Fig. 2E, F) compared with the control (Fig. 2C, D). Embryos injected with the *smyd1a* E9I9-MO, however, showed small head and developmental delay, suggesting some off-target toxic effect. The off-target toxic effect could be alleviated by co-injection with the p53-MO (Fig. 2G, H). To confirm that knockdown of Smyd1a did not affect early muscle development, we analyzed the specification of slow and fast muscle precursors and their subsequent migration and differentiation. Compared with control (Fig. 2I), MyoD expression appeared normal in slow and fast muscle precursors of *smyd1a* knockdown embryos (Fig. 2I, J). In addition, fiber-type-specific expression of slow or fast myosin heavy chain (MHC) genes also appeared normal (Fig. 2M, N). Slow fibers were clearly localized at the superficial layer of the myotome in *smyd1a* knockdown embryos (Fig. 2M), suggesting that migration of slow muscle cells was not affected by *smyd1a* knockdown.

### 4. Knockdown of *smyd1a* and *smyd1b* together Resulted in a Stronger Muscle Phenotype in Zebrafish Embryos

**Figure 3:**
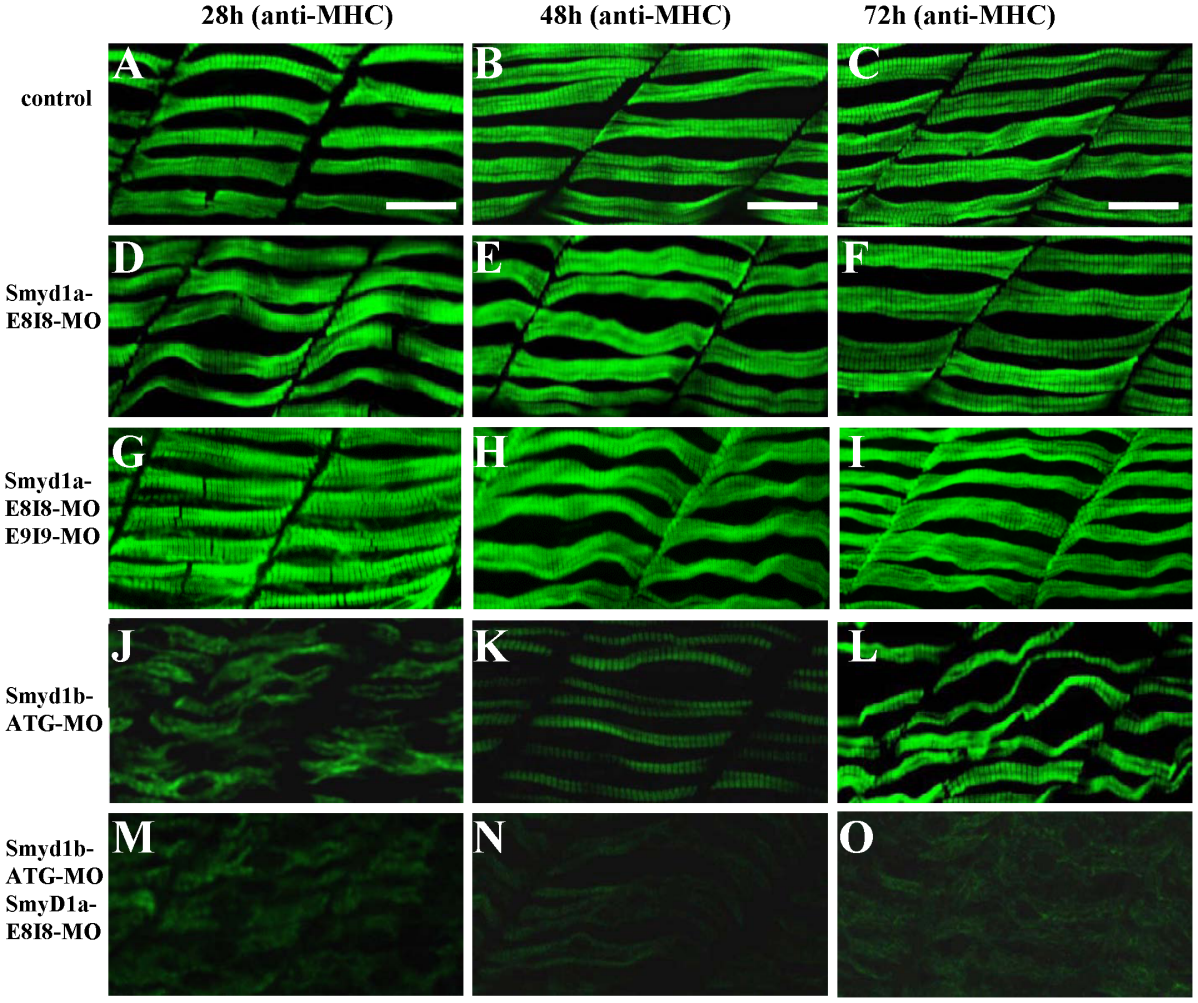
The effect of *smyd1a* and/or *smyd1b* knockdown on myosin thick filament organization in skeletal muscles. Zebrafish embryos injected with *smyd1b* MO or *smyd1a* MO or both were fixed at 28, 48 and 72 hpf. Myosin thick filament organization was analyzed by immunostaining with the F59 antibody which recognizes the myosin heavy chain (MHC) in slow muscles, and followed by FTIC-labeled secondary antibody. The images represent side view of trunk muscles around segment 10. **A–C.** Lateral view of thick filament organization in slow muscle fibers of control-MO injected embryos at 28 (A), 48 (B) and 72 (C) hpf. **D–F.** Lateral view of thick filament organization in slow muscle fibers of *smyd1a* E8I8-MO injected embryos at 28 (D), 48 (E) and 72 (F) hpf. **G–I.** Lateral view of thick filament organization in slow muscle fibers of *smyd1a* E8I8-MO and E9I9-MO co-injected embryos at 28 (G), 48 (H) and 72 (I) hpf. **J–L.** Lateral view of thick filament organization in slow muscle fibers of *smyd1b* ATG-MO injected embryos at 28 (J), 48 (K) and 72 (L) hpf. **M–O.** Lateral view of thick filament organization in slow muscle fibers of *smyd1a* E8I8-MO and *smyd1b* ATG-MO co-injected embryos at 28 (M), 48 (N) and 72 (O) hpf. Scale bars: 20 µm in A–C.

To analyze the effects of *smyd1a* knockdown on sarcomere assembly in skeletal muscles, we first examined the organization of thick filaments in slow myofibers of *smyd1a* knockdown zebrafish embryos. The results showed that knockdown of *smyd1a* alone had no detectable effect on the sarcomere organization of the myosin thick filaments (Fig. 3D–F) in slow fibers of zebrafish embryos at 24, 48 and 72 hpf. The lack of muscle defect from *smyd1a* knockdown could be due to the inefficient knockdown. To determine whether increasing the efficiency of *smyd1a* knockdown could affect muscle development, we co-injected the two *smyd1a* splicing MOs into zebrafish embryos. Compared with the single MO injection, the results showed that co-injection of the two MOs increased the efficiency of *smyd1a* knockdown (Fig. 2B). However, it did not result in more defects on myofibril assembly in slow muscles (Fig. 3G–I).

To test the possibility that the lack of muscle defect from *smyd1a* knockdown was due to the redundant function from *smyd1b*, we carried out the double knockdown of *smyd1a* and *smyd1b* in zebrafish embryos. The data showed that knockdown of *smyd1a* and *smyd1b* together resulted in a stronger muscle phenotype compared with *smyd1a* or *smyd1b* knockdown alone. As shown in Fig. 3J, knockdown of *smyd1b* alone resulted in disruption of myosin thick filament organization at 28 hpf. However, the defective thick filament organization was partially recovered in *smyd1b* knockdown zebrafish embryos at 48 and 72 hpf (Fig. 3K, L). Double knockdown studies showed that the partial recovery from *smyd1b* knockdown was diminished when *smyd1b* was knocked down together with *smyd1a* (Fig. 3N,O), suggesting that Smyd1a may have a partial function redundancy with Smyd1b in myofibril assembly.

**Figure 4:**
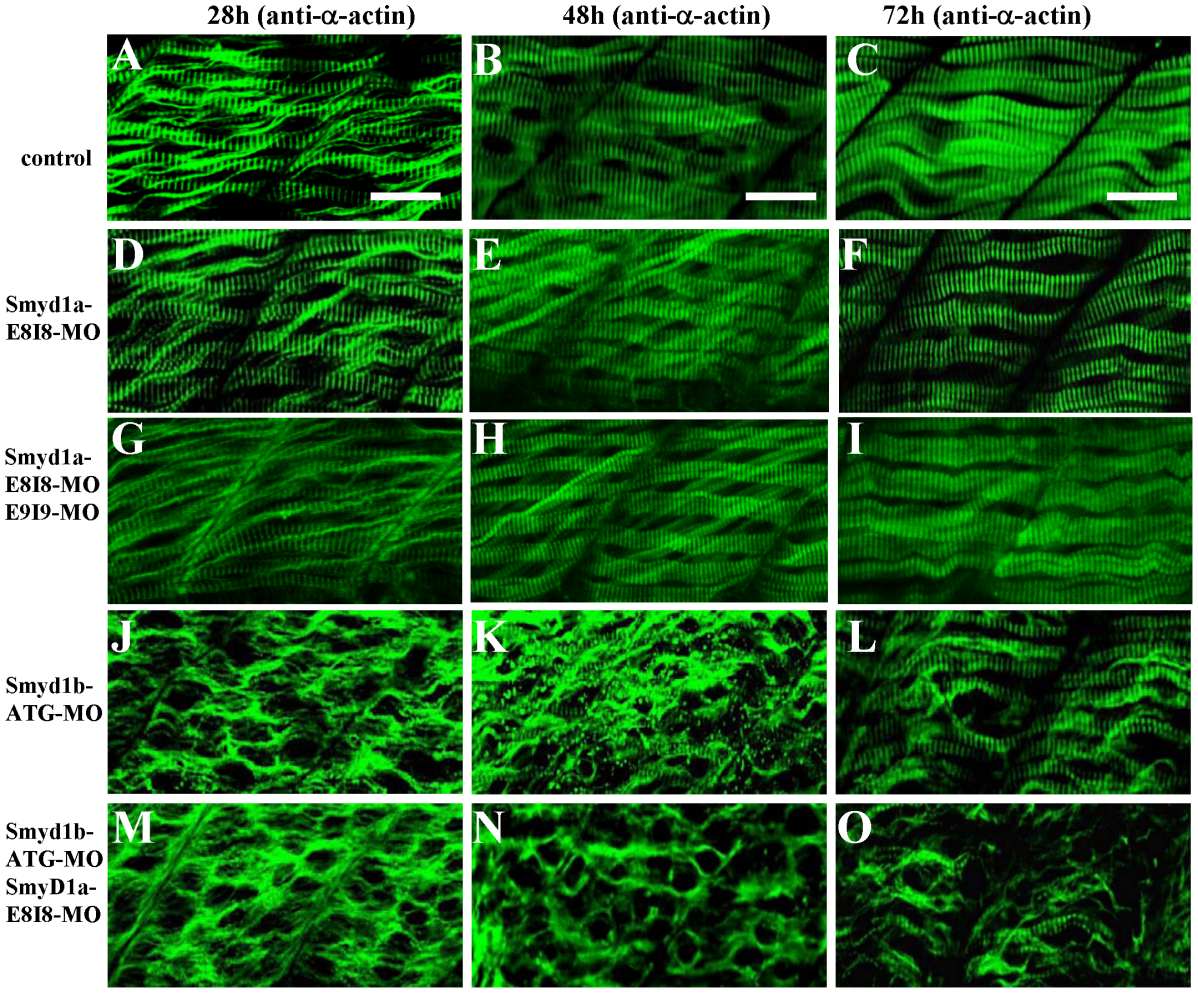
The effect of *smyd1a* or *smyd1b* single or double knockdown on thin filament organization in skeletal muscles. Zebrafish embryos injected with *smyd1b* MO or *smyd1a* MO or both were fixed at 28, 48 and 72 hpf. α-actin thin filament organization was analyzed by immunostaining with anti-α-actin antibody (Acl-20.4.2), and followed by FTIC-labeled secondary antibody. The images represent side view of trunk muscles around segment 10. **A–C.** Lateral view of thin filament organization in skeletal muscle fibers of control-MO injected embryos at 28 (A), 48 (B) and 72 (C) hpf. **D–F.** Lateral view of thin filament organization in skeletal muscle fibers of *smyd1a* E8I8-MO injected embryos at 28 (D), 48 (E) and 72 (F) hpf. **G–I.** Lateral view of thin filament organization in slow muscle fibers of *smyd1a* E8I8-MO and E9I9-MO co-injected embryos at 28 (G), 48 (H) and 72 (I) hpf. **J–L.** Lateral view of thin filament organization in skeletal muscle fibers of *smyd1b* ATG-MO injected embryos at 28 (J), 48 (K) and 72 (L) hpf. **M–O.** Lateral view of thin filament organization in skeletal muscle fibers of *smyd1a* E8I8-MO and *smyd1b* ATG-MO co-injected embryos at 28 (M), 48 (N) and 72 (O) hpf. Scale bars: 20 µm in A–C.

**Figure 5:**
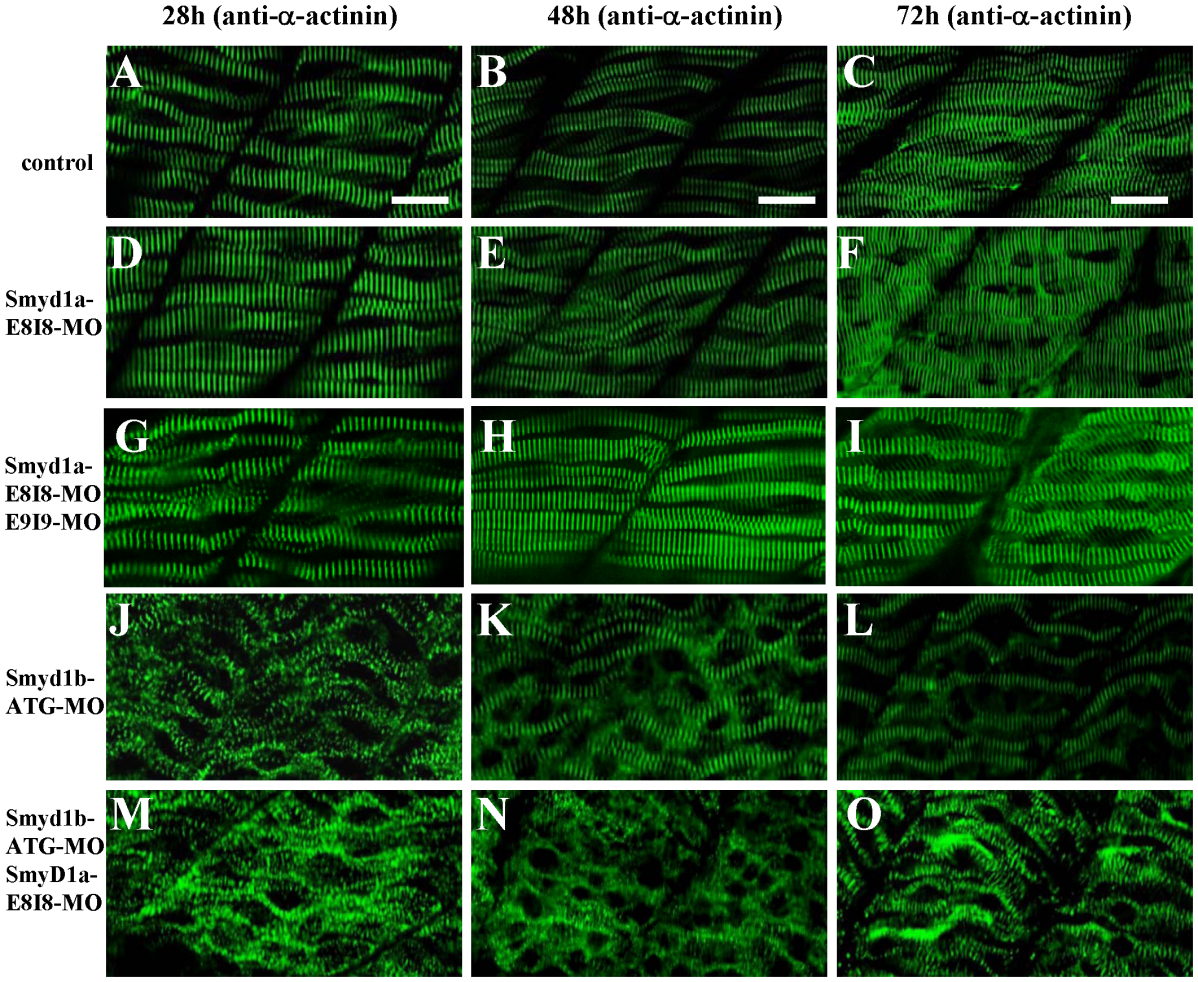
The effect of *smyd1a* or *smyd1b* single or double knockdown on the Z-line organization in skeletal muscles. Zebrafish embryos injected with *smyd1b* MO or *smyd1a* MO or both were fixed at 28, 48 and 72 hpf. Z-line organization was analyzed by immunostaining with anti-α-actinin antibody (EA-53), and followed by FTIC-labeled secondary antibody. The images represent side view of trunk muscles around segment 10. **A–C.** Lateral view of Z-line organization in skeletal muscle fibers of control-MO injected embryos at 28 (A), 48 (B) and 72 (C) hpf. **D–F.** Lateral view of Z-line organization in skeletal muscle fibers of *smyd1a* E8I8-MO injected embryos at 28 (D), 48 (E) and 72 (F) hpf. **G–I.** Lateral view of Z-line organization in skeletal muscle fibers of *smyd1a* E8I8-MO and E9I9-MO co-injected embryos at 28 (G), 48 (H) and 72 (I) hpf. **J–L.** Lateral view of Z-line organization in skeletal muscle fibers of *smyd1b* ATG-MO injected embryos at 28 (J), 48 (K) and 72 (L) hpf. **M–O.** Lateral view of Z-line organization in skeletal muscle fibers of *smyd1a* E8I8-MO and *smyd1b* ATG-MO co-injected embryos at 28 (M), 48 (N) and 72 (O) hpf. Scale bars: 20 µm in A–C.

To determine whether this was also true for other sarcomeric structures, we analyzed the α-actin thin filaments and Z-line organization in the single or double knockdown embryos. The results showed that the knockdown of *smyd1a* and *smyd1b* together had a stronger effect on thin filament disruption at 72 h pf (Fig. 4O) compared with the knockdown alone (Fig. 4I, L). A similar finding was also observed with the Z-line structure (Fig. 5). Collectively, these data indicate that Smyd1a and Smyd1b are required for myofibril organization and assembly in skeletal muscles of zebrafish embryos although Smyd1b plays a dominant role compared with Smyd1a.

**Figure 6:**
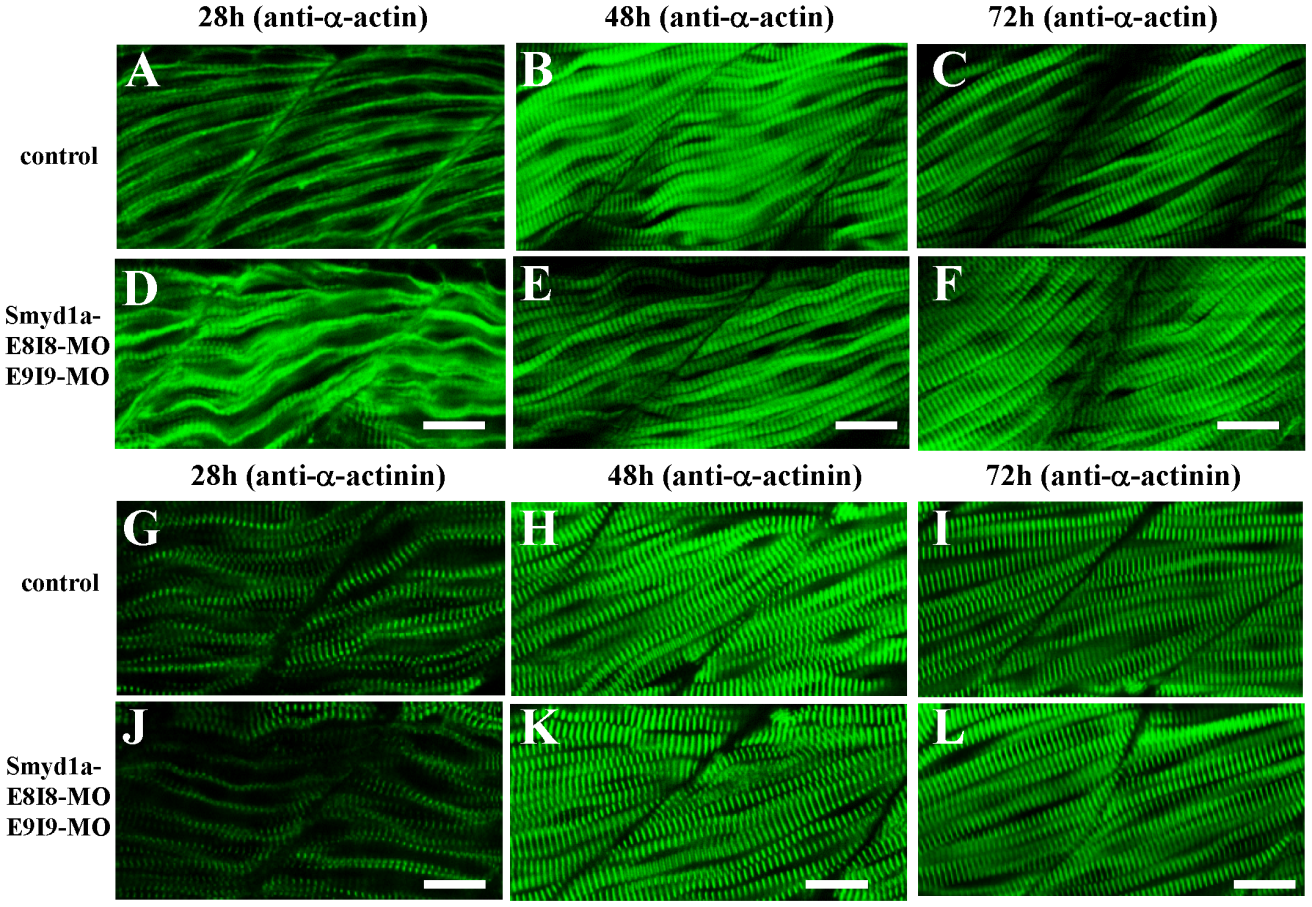
The effect of *smyd1a* knockdown on the sarcomere organization in fast muscles. Zebrafish embryos co-injected with *smyd1a* E8I8 and E9I9 MOs were fixed at 28, 48 and 72 hpf. The sarcomere organization of thin filaments and Z-lines was analyzed by immunostaining with anti-α-actin and anti-α-actinin antibody, respectively, and followed by FTIC-labeled secondary antibody. The images represent side view of trunk muscles around segment 10. **A–C.** Lateral view of thin filament organization in skeletal muscle fibers of control-MO injected embryos at 28 (A), 48 (B) and 72 (C) hpf. **D–F.** Lateral view of thin filament organization in skeletal muscle fibers of *smyd1a* MO injected embryos at 28 (D), 48 (E) and 72 (F) hpf. **G–I.** Lateral view of Z-line organization in skeletal muscle fibers of control-MO injected embryos at 28 (G), 48 (H) and 72 (I) hpf. **J–L.** Lateral view of the Z-line organization in skeletal muscle fibers of *smyd1a* MO injected embryos at 28 (J), 48 (K) and 72 (L) hpf. Scale bars: 20 µm in D–F and J–L.

In situ expression analysis showed that *smyd1a* was expressed in fast muscles (Fig. 1). To determine whether knockdown of *smyd1a* had any effect on fast muscles, sarcomere organization was characterized in fast muscles of *smyd1a* knockdown embryos. The results showed that knockdown of *smyd1a* alone had no effect on myofibril assembly in fast muscles (Fig. 6).

### 5. Ectopic Expression of Zebrafish *smyd1a* or Mouse *Smyd1* Transgene Could Rescue the Myofibril Defects in *smyd1b* Knockdown Zebrafish Embryos

It has been suggested that actinopterygian fish, such as zebrafish, had gone through one more round of whole genome duplication compared with mammals during evolution [18]. The two *Smyd1* genes in zebrafish were likely generated by the gene duplication event in teleosts because mouse and human genomes contain only one *Smyd1* gene. Sequence analysis revealed that zebrafish Smyd1a and Smyd1b share high sequence similarity with the mouse and human Smyd1 (Fig. S2). However, functional studies revealed that knockdown of *smyd1a* or *smyd1b* resulted in strong differences in muscle phenotype. Knockdown of *smyd1b* significantly disrupted the myofibril organization, whereas knockdown of *smyd1a* alone had little effect. The different severity of the knockdown phenotypes could be due to structural changes of *smyd1a* protein that made it less active compared with *smyd1b*. Alternatively, the different knockdown phenotype could be caused by their distinct patterns of temporal expression because *smyd1a* was expressed several hours later than *smyd1b* in zebrafish embryos.

**Figure 7:**
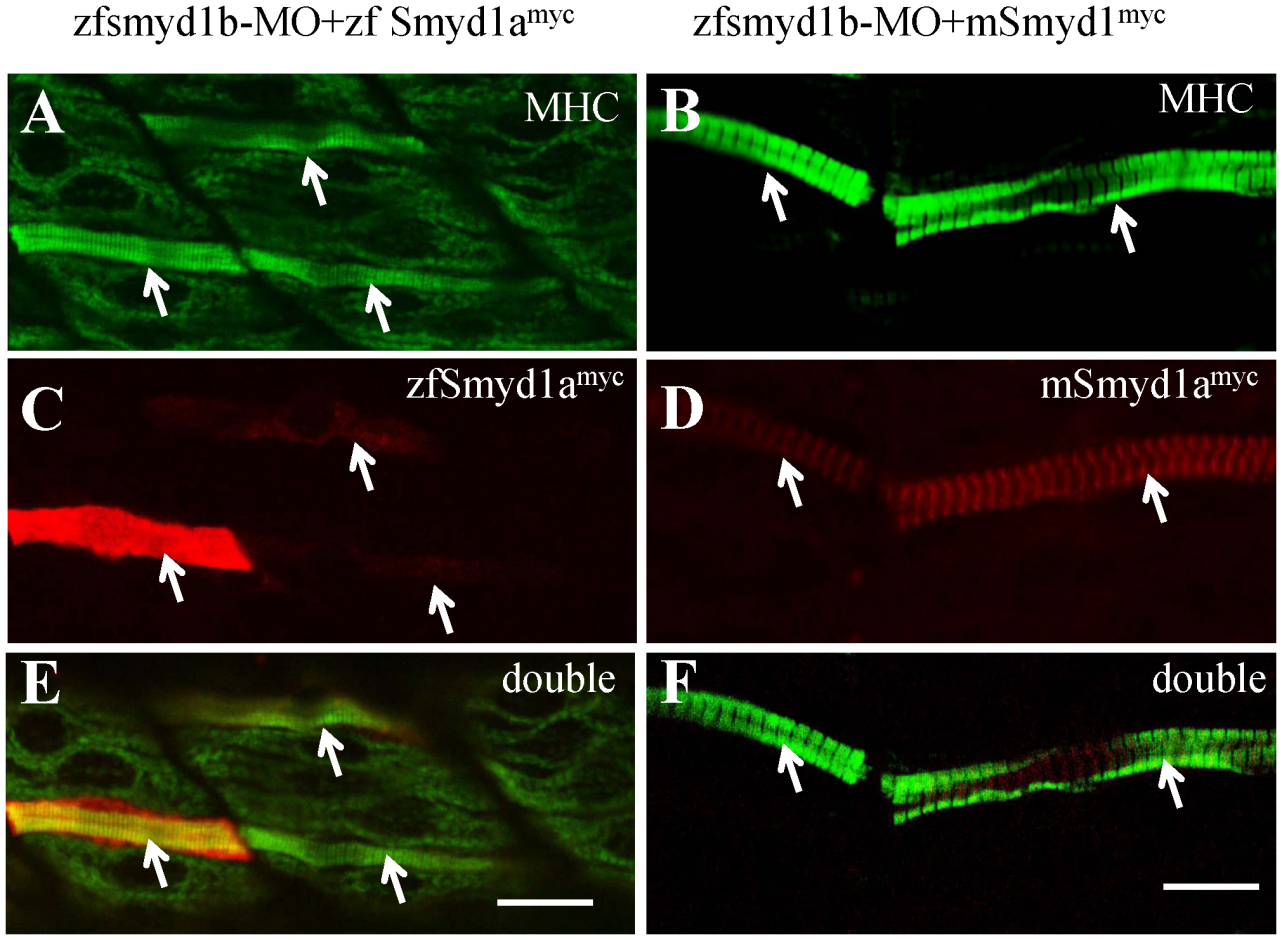
The myofibril defects from *smyd1b* knockdown could be rescued by ectopic expression of zebrafish *smyd1a* or mouse Smyd1. The *smyd1b* ATG-MO was co-injected with *smyd1b:zfsmyd1a^myc^* or *smyd1b:mSmyd1^myc^* transgene into zebrafish embryos at 1 or 2 cells stages. Myosin thick filament organization and transgene expression were analyzed by double immunostaining with anti-MHC (F59, green) and anti-myc (9E10, red) antibodies. **A, C, E.** Double immunostaining with anti-MHC (A) and anti-myc (C) antibodies shows the normal thick filament organization in myofibers expressing the myc-tagged zebrafish *smyd1a* transgene at 24 hpf. E, merged picture of **A** and **C. B, D, F.** Double immunostaining with anti-MHC (B) and anti-myc (D) antibodies shows the normal thick filament organization in myofibers expressing the myc-tagged mouse Smyd1 transgene at 24 hpf. F, merged picture of B with D. D and F showed the sarcomeric localization of myc-tagged mouse Smyd1 (red). The myc-tagged mouse Smyd1 was localized in the middle of the MHC thick filament (F), a region normally occupied by the M-line. Scale bars: 20 µm in E; 14 µm in F.

To clarify these questions, we tested whether ectopic expression of *smyd1a* early in zebrafish embryos could rescue the *smyd1b* knockdown phenotypes. A rescue experiment was performed in the *smyd1b* knockdown zebrafish embryos using the *smyd1a* transgene (*Smyd1-zfsmyd1a^myc^*) directed by the *smyd1b* promoter. The *Smyd1-zfsmyd1a^myc^* transgene expressing a myc-tagged zebrafish Smyd1a was co-injected with *smyd1b* ATG-MO into zebrafish embryos. The *smyd1b* ATG-MO could specifically knock down the expression of *smyd1b*, however it had no effect on the expression of the zebrafish *smyd1a^myc^* transgene because the transgene did not contain the *smyd1b* ATG-MO target sequence. Myofibril organization and ectopic Smyd1a^myc^ expression was analyzed in the co-injected zebrafish embryos by double staining with anti-MyHC (F59) and an anti-myc antibodies. The data showed a clear rescue of thick filament organization in the co-injected embryos (Fig. 7A). The rescue appeared in a mosaic pattern, consistent with the pattern of gene expression through DNA injection. Double staining revealed a perfect match between the rescued myofibers and the expression of the myc-tagged zebrafish Smyd1a (Fig. 7C, E). Collectively, these data indicate that Smyd1a could replace Smyd1b function if expressed early in muscle cells, arguing that the different severity of *smyd1a* and *smyd1b* knockdown phenotypes was likely due to distinct pattern of temporal expression.

Sequence analysis revealed that Smyd1a and Smyd1b share similar sequence identity with mouse and human Smyd1 (Fig. S2). To test whether Smyd1 function is conserved during evolution, we performed a rescue experiment in the *smyd1b* knockdown zebrafish embryos using the mouse *smyd1* transgene directed by the zebrafish *smyd1b* promoter. The mouse *Smyd1* transgene (*smyd1-mSmyd1^myc^*) expressing a myc-tagged mouse Smyd1 was co-injected with the *smyd1b* ATG-MO into zebrafish embryos. Double staining revealed that expression of the myc-tagged mouse Smyd1 could rescue the myofibril defects from the *smyd1b* knockdown in a cell autonomous manner (Fig. 7B, D, F). Collectively, these data indicate that Smyd1function in myofibril organization is likely conserved during evolution.

### 6. The Subcellular Localization of Zebrafish Smyd1a and Mouse Smyd1 in Myofibers

Previous studies by us and others have shown that Smyd1b is localized on the M-line of sarcomeres in both skeletal and cardiac muscles [6], [16]. This was especially evident for the longer isoform Smyd1b_tv1 [16]. It is not clear whether zebrafish Smyd1a and mouse Smyd1 could also localize on M-lines of sarcomere in skeletal muscles. To clarify this question, we examined the subcellular localization of myc-tagged zebrafish Smyd1a and mouse Smyd1 in skeletal muscles of zebrafish embryos. The results showed that, similar to Smyd1b, mouse Smyd1 was localized on the sarcomere of skeletal muscles (Fig. 7D). However, unlike Smyd1b, zebrafish Smyd1a showed no clear sarcomeric localization in myofibers (Fig. 7C).

**Figure 8:**
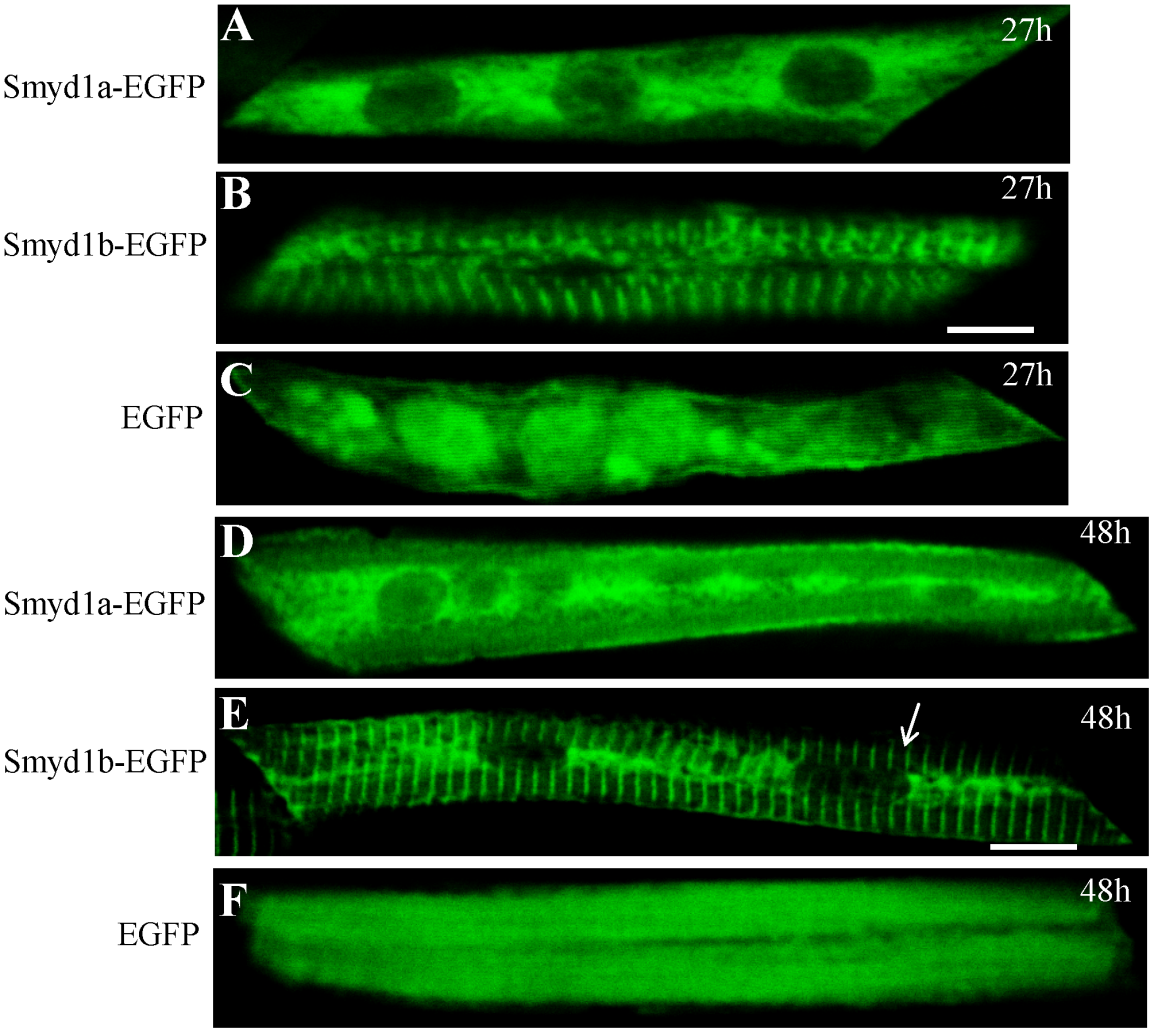
Characterization of the Smyd1a subcellular localization using the Smyd1a-EGFP fusion protein. DNA constructs expressing the zebrafish Smyd1a-EGFP or Smyd1b-EGFP fusion proteins or EGFP control were injected into zebrafish embryos. Their expression and localization were determined in myofibers of the injected zebrafish embryos at 27 (A, B, C) and 48 (D, E, F) hpf. A and D represent Smyd1b-EGFP; B and E represent Smyd1a-EGFP; C and F represent EGFP control. Scale bars: 6 µm in B; 10 µm in E.

To enhance the sensitivity of the detection, we generated a transgene, *pTol2-Smyd1a -EGFP*, expressing a GFP-tagged zebrafish Smyd1a fusion protein. The *pTol2-Smyd1a-EGFP* transgene was injected into zebrafish embryos. Smyd1a-EGFP expression and subcellular localization were characterized in the injected zebrafish embryos by direct observation. The results showed that Smyd1b-EGFP had a clear sarcomeric localization (Fig. 8B, E). In contrast, Smyd1a-EGFP was not localized on the sarcomeres (Fig. 8A, D). A diffuse cytosolic localization was detected for Smyd1a-EGFP in myofibers of zebarfish embryos (Fig. 8A, B), similar to EGFP control (Fig. 8C, F). Together, these data indicate that Smyd1a and Smyd1b have distinct subcellular localization, indicating that the sarcomeric localization of Smyd1b might not be directly linked with the Smyd1 function in sarcomere organization because Smyd1a could rescue the myofibril defects from Smyd1b knockdown although Smyd1a was not localized on the sarcomere. This is consistent with previous findings that either one of the Smyd1b isoforms, Smyd1b_tv1 or Smyd1b_tv2, from alternative splicing was able to rescue the *smyd1b* knockdown phenotype although their proteins showed distinct subcellular localization [5], [16].

## Discussion

In this study, we have characterized the muscle phenotype from knockdown of *smyd1a* or *smyd1b* alone, or together in zebrafish embryos. We demonstrated that in contrast to *smyd1b* which is absolutely required for myofibril organization in skeletal muscles, knockdown of *smyd1a* alone had very little effect on myofibril organization. However, knockdown of *smyd1a* and *smyd1b* together resulted in a stronger muscle phenotype compared with knockdown of *smyd1a* or *smyd1b* alone. We further demonstrated that the muscle defects from *smyd1b* knockdown could be rescued by the ectopic expression of the zebrafish *smyd1a* or mouse *Smyd1,* suggesting that Smyd1 function is likely to be conserved during evolution. Finally, we showed that similar to Smyd1b, mouse Smyd1 was localized on sarcomeres in skeletal myofibers. In contrast, zebrafish Smyd1a did not show any sarcomeric localization although it could rescue the Smyd1b knockdown defects.

### The Unequal Role of Smyd1a and Smyd1b in Myofibril Assembly

Gene duplication plays a vital role in evolution [19]. Sequence analysis revealed that zebrafish genome contains two *smyd1* genes, *smyd1a* and *smyd1b* that were likely generated by the whole genome duplication in actinopterygian fish during evolution [17], [18], [20]. *smyd1a* and *smyd1b* are paralogous genes that are dispersed on different chromosomes in zebrafish. Sequence analysis revealed that zebrafsh *smyd1a* and mouse *Smyd1* showed a highly conserved synteny arrangement with the surrounding THNSL2 and FABP1 genes. The synteny arrangement with *fabp1*was maintained for *smyd1b* in zebrafish. However, this synteny was not found between the zebrafish *smyd1b* and *thnsl2* genes, suggesting that a genomic rearrangement or deletion might occur in this region after the whole genome duplication.

It has been suggested that after whole genome duplication, the duplicated genes could evolve in several directions without the selective pressure. Some of the duplicated genes could gain new functions, while many others turned into nonfunctional pseudogenes [19], [20]. We showed in this study that both *smyd1a* and *smyd1b* are functional genes after the duplication. However, they do not play an equal role in myofibril organization. Smyd1b appears to be more critical than Smyd1a because knockdown of *smyd1b* gave a stronger muscle phenotype compared with *smyd1a* knockdown. Our data indicate that functional difference between Smyd1a and Smyd1b could be caused by their different patterns of temporal expression, different activity due to changes of protein structure, or different levels of gene expression. We showed that early ectopic expression of *smyd1a* by using the *smyd1b* promoter could rescue the myofibril defects from *smyd1b* knockdown, suggesting that Smyd1a could function as Smyd1b if expressed in a similar temporal and spatial pattern in zebrafish embryos. At present, it is not clear whether Smyd1a gained new functions during evolution. Knockdown of *smyd1a* alone failed to reveal any significant phenotype in zebrafish embryos, unless it was knocked down together with *smyd1b*. Together, these studies suggest that Smyd1a is involved in myofibril assembly, but with a minor role in the process.

### The Smyd1 Function in Myofibril Organization is Conserved during Evolution

Our studies demonstrated that Smyd1 function in myofibril organization is likely to be conserved during evolution because ectopic expression of mouse *Smyd1* could rescue the muscle defects from *smyd1b* knockdown in zebrafish embryos. Unlike zebrafish, there is only one *Smyd1* gene in mouse and human genomes. Consistent with the idea that zebrafish *smyd1b* is the ortholog of *Smyd1* in mice, it has been reported that knockout of *Smyd1* in mice resulted in early embryonic lethality from cardiomyogenesis defects [4]. A cardiac muscle defect was also observed in Smyd1b knockdown or mutant zebrafish embryos [5], [6]. In addition to their functional similarity, the zebrafish Smyd1b and mouse Smyd1 share other common features in gene expression and protein localization. First, both zebrafish *smyd1b* and mouse *smyd1* express two alternatively spliced mRNA transcripts that differ by 39 bp encoded by exon 5. Second, both mouse Smyd1 and zebrafsh Smyd1b are localized on the M-lines of sarcomeres in skeletal and cardiac muscles [6], which is in contrast to the zebrafish Smyd1a that showed no sarcomeric localization.

### The Sarcomeric Localization of Smyd1

The biological significance of the sarcomeric localization is not clear. It has been reported that Smyd1b could bind to myosin and showed a transient localization with thick filaments during myofibrillogenesis in zebrafish embryos [6]. However, after the completion of sarcomere organization, the Smyd1b is translocated to the M-line. Interesting, Etard and colleagues have shown that myosin chaperones Unc45b and Hsp90α could shuttle between the A band and the Z line in response to stress or damage to the myofiber [21]. It has been suggested that the sarcomeric localization of myosin chaperones and their translocation within different parts of the muscle cells could be involved in the response of muscle cells to mount efficient physiological responses to muscle stress, load requirements, and/or stretch [21]. It remains to be determined whether the sarcomeric localization of Smyd1b is involved in sarcomere remodeling and response to muscle stress.

We have shown previously that the sarcomeric localization of Smyd1b requires Phe223 and Serine 225 [16]. Substitution of Phe223 or Ser225 with alanine significantly diminished the sarcomeric localization of Smyd1b [16]. Interestingly, the two highly conserved residues Phe223 and Ser225 are present in zebrafish Smyd1a, arguing that in addition to the Phe223 and Se225 residues, other residues or motifs are likely to be involved in the sarcomeric localization. Sequence analysis revealed that Smyd1a differs from Smyd1b significantly at the C-terminal sequence (Figure S1). Compared with Smyd1a, Smyd1b has seven extra amino acids at the C-terminus including three highly conserved residues (xxLFxxK) that are present in all Smyd1 proteins that have been identified in vertebrates. It remains to be determined whether these residues may contribute to the sarcomeric localization of Smyd1b.

## Materials and Methods

### Ethics Statement

This study was carried out in strict accordance with the recommendations in the Guide for the Care and Use of Laboratory Animals of the National Institutes of Health. The protocol was approved by the Institutional Animal Care and Use Committee of the University of Maryland (Permit Number:0610009).

### Fish Maintenance and Use

Zebrafish were maintained at 28.5 C in 10 gallon aquarium supplied with fresh water and air at a photoperiod of 14 hours of light and 10 hours of dark at the Zebrafish Facility at Aquaculture Research Center (ARC) in Columbus Center (Baltimore). Pairs of adult male and female zebrafish were put in 1 liter tanks for natural spawning after lights were turned on in the facility. Embryos were collected and raised at 28.5 C. To ease pain and facilitate animal handling, fish embryos over one day old were anaesthetized in 0.6 mM Tricaine that has been buffered to neutral pH around 7. The anaesthetized embryos were sacrificed quickly by keeping on ice and used directly for RNA extraction or fixation for immunostaining. All procedures were carried out in compliance with the guidelines stipulated by the Institutional Animal Care and Use Committee of the University of Maryland.

### Isolation of Zebrafish smyd1a cDNA

The full-length coding sequence of zebrafish *smyd1a* (NM_205540) was cloned by RT-PCR using total RNA extracted from zebrafish embryos at 24–72 hpf. The PCR was carried with the Advantage PCR kit using the following primers: zf*smyd1a*-P1 (5′ –AGCATGACCGTGGAGAAGACGGAC-3**′**) and zf*smyd1a*-P2 (5**′** –GTGTTCATGCTTTGATCTGCACTT-3**′**) derived from DNA sequences near the start codon and stop codon, respectively. The PCR product was cloned into pGEM-T vector. The resulting plasmid was named pGEM-T-zf*smyd1a*.

### Analysis of smyd1a and Smd1b Expression in Zebrafish Embryos by RT-PCR

To analyze the temporal pattern of *smyd1a* expression during development, total RNA was extracted from zebrafish embryos at 20 min, 3h, 6h, 9h, 12h, 16h, 1d, 2d, 3d, 4d and 5d post fertilization. *smyd1a* expression was determined by RT-PCR using zf*smyd1a*-P1 and zf*smyd1a*-P2 primers. To characterize the expression of tv1 and tv2 isoforms from alternative splicing of exon 5, RT-PCR was carried out using two new sets of primers *smyd1a*-tv-P1/P2 or *smyd1b*-tv-P1/P2, respectively. PCR using these primers produced small PCR fragments covering the alternatively spliced exon 5.

*smyd1a*-tv-P1∶5**′**-GTCTGAATCGGTCGGCATCGGC-3′.

*smyd1a*-tv-P2∶5′-CATAACTGACGGTCAGCTCCTGT-3′.

*smyd1b*-tv-P1∶5′-TCATGGTGAGCGATCAGCGCGGC-3′.

*smyd1b*-tv-P2∶5′-ACGTTCAGATAATCCACATACGC-3′.

### Synthesis of Morpholino Antisense Oligos

Morpholino antisense oligos were synthesized by Gene Tools. The *smyd1b* ATG-MO was based on the target sequence surrounding the ATG start codon. The two *smyd1a* splicing blockers (E8I8-MO, E9I9-MO) were based on the sequence at the exon-8/intron-8 or exon-9/intron-9 junction, respectively. The standard control MO from Gene Tools was used as control.

*smyd1b* ATG-MO: 5′ - ACTTCCACAAACTCCATTCTGGATC-3′.

*smyd1a* E8I8-MO: 5′ - ATATCGCAACACTCACATGTATCCA-3′.

*smyd1a* E9I9-MO: 5′ - GGTTGTACCTCCAGGTCTCTGCTGA-3′.

### Morpholino and DNA Microinjection in Zebrafish Embryos

Morpholino antisense oligos were dissolved in 1x Danieau buffer [22] to a final concentration of 0.5 mM. Approximately 1–2 nl (5–10 ng) of MO was injected into each embryo at 1 or 2 cell stages. For co-injection with transgenes, equal volumes of MO (1 mM) and DNA construct (100 µg/ml) was mixed and 1–2 nl was microinjected into each embryo at 1 or 2 cell stages. All the microinjection experiments and subsequent analyses were carried out at least three times with 100–150 embryos per sample in each experiment.

### Analysis of Smyd1a mRNA Splicing in MO Injected Embryos by RT-PCR

To determine the efficacy of E8I8-MO and E9I9-MO splicing blocker on *smyd1a* transcript splicing, total RNA was extracted from E8I8-MO or E9I9-MO single injection or E8I8-MO and E9I9-MO co-injected embryos at 48 hpf. *smyd1a* transcripts were amplified using *smyd1a*-E7-F1 and *smyd1a*-P2 primers or *smyd1a*-E7/8-F1 and *smyd1a*-P2 primers. The *smyd1a*-E7-F1 primer (5′ - CTTCAGGCTCTGGTGAAGATTGAA-3′) was derived from the exon7. The *smyd1a*-E7/8-F1 primer (5′ -ACTTCCATGAGGTGATCAGGATCTG-3′) was derived from the junction of the exon7 and exon 8 sequences, while the *smyd1a*-P2 primer (5′ -TCATGCTTTGATCTGCACTTG- 3′) was from the antisense sequence near the stop codon. The PCR products were cloned into pGEM-T Easy vector for sequencing analysis.

### Construction of Zebrafish smyd1b-zfsmyd1a^myc^ Transgene

To generate the *smyd1b*-zf*smyd1a* ^myc^ transgene for muscle specific expression of myc-tagged zebrafish Smyd1a, the coding sequence of the zebrafish *smyd1a* was amplified with pfu DNA polymerase using the pGEM-T-zf*smyd1a* plasmid as a template. The PCR was carried out using zf*smyd1a*-P1 and zf*smyd1a*-3′-myc primers. The zf*smyd1a*-P1 primer (5′-AGCATGACCGTGGAGAAGACGGAC- 3′) contained the ATG start codon. The zf*smyd1a*-3′-myc primer (5′-CTAATTCAGGTCCTCTTCAGAGATGAGCTTCTGCTCTGCTTTGATCTGCACTTGTTC- 3′) contained a myc-tag coding sequence and a stop codon. The PCR product containing the full-length coding sequence of a myc-tagged zebrafish *smyd1a* was phosphorylated with T4 kinase and directly cloned in the SmaI sites of the muscle-specific *smyd1b-gfp* vector [9]. The resulting plasmid was named *smyd1b*-zf*smyd1a* ^myc^.

### Construction of smyd1b-mSmyd1^myc^construct

Mouse *smyd1a* cDNA was cloned by RT-PCR using total RNAs from adult mouse skeletal muscles. The PCR was carried with the Advantage PCR kit using the mouse mSmyd1-ATG (BH1) and mSmyd1–3′-myc primers. The PCR product was cloned into pGEM-T vector. The resulting plasmid was named pGEM-T-m*smyd1a* ^myc^.

The mSmyd1-ATG (BH1) primer (5′-GGATCCATGGAGAACGTGGAGGTCTTC- 3′) contained the ATG start codon, while the mSmyd1–3′-myc primer (5′CTAATTCAGGTCCTCTTCAGAGATGAGCTTCTGCTCCTGCTTCTTATGGAACAGAG-3′) contained the myc-tag coding sequence and a stop codon.

To generate the *smyd1b*-*mSmyd1^my^* ^c^ transgene for muscle specific expression of myc-tagged mouse Smyd1, the coding sequence of a myc-tagged mouse Smyd1 was amplified from the pGEM-T-mSmyd1^myc^ plasmid by pfu DNA polymerase. The PCR was carried using the following primers: mouse Smyd1-ATG (BH1) and mouse Smyd1-3′-myc. The PCR product containing the coding sequence of mouse Smyd1 and the myc-tag at the C-terminus was phosphorylated with T4 kinase and directly cloned into the SmaI sites of the muscle-specific *smyd1b* vector [9]. The resulting plasmid was named *smyd1b*-*mSmyd1^myc^.*

### Construction of Tol2-zfsmyd1a-EGFP Construct

The pTol2-*smyd1a*-EGFP construct was generated by cloning the zebrafish *smyd1a* coding sequence in frame upstream of the EGFP coding sequence in the T2A200R150G vector [23]. Briefly, the zebrafish *smyd1a* coding sequence without the stop codon were generated by PCR using pfu DNA polymerase (Stratagene). The PCR was carried out using the *smyd1b-zfsmyd1a^myc^* plasmid as a template together with the zf*smyd1a*-F (5′- gctAGATCTatgaccgtggagaagacggaccc-3′) and zf*smyd1a*-R (5′- gctAGATCTgctttgatctgcacttgttctcc-3′) primers. A *BglII* site was introduced at the 5′ and 3′ ends of the *smyd1a* coding sequence via the respective PCR primers. The PCR products were digested with *BglII* and then cloned into the compatible *BamHI* site of the T2A200R150G vector. The DNA sequence at the *smyd1a* and EGFP junction was confirmed by sequencing.

### Whole Mount in Situ Hybridization and Immunostaining

Whole mount in situ hybridization was carried out using digoxigenin-labeled antisense probes as previously described [24]–[26]. The pGEM-T-zf*smyd1a* plasmid was digested with NcoI and transcribed with Sp6 RNA polymerase to synthesize digoxigenin-labled antisense RNA probes. Immunostaining was carried out using whole mount zebrafish embryos (1–3 dpf) as previously described [5], [27]. Briefly, zebarfish embryos were fixed in 4% paraformaldehyde (in PBS) for 1 hour at room temperature. The fixed embryos were washed for15 minutes 3 times in PBST (0.5% Tween-20 in 1x PBS). Embryos of 48 and 72 hpf were digested with 1 mg/ml collagenase for 45 and 75 minutes, respectively. In situ hybridization for MyoD, slow and fast myosin heavy chains were carried out using the respective digoxigenin-labeled probes as descried previously [28].

Immunostaining was performed with the following primary antibodies: anti-α-actinin (clone EA-53, #A7811, Sigma), anti-MHC for slow muscles (F59, DSHB), anti-actin (Ac1–20.4.2, Progen), and anti-myc tag (71D10, Cell Signaling). Secondary antibodies were FTIC or TRIC-conjugates (Sigma). The images were acquired using an upright microscope (Zeiss, Oberkochen, Germany) equipped with a confocal image analyzer (BIO-RAD Radiance 2100 Imaging Systems, Hercules, CA).

## Supporting Information

Figure S1 **Structure comparison of zebrafish Smyd1a and Smyd1b. A.** The synteny arrangement of zebrafish *smyd1b* and mouse *smyd1* with *fatty acid binding protein 1b* (*fabp1b*) and *thronine synthase like 2* (*thnsl2*) genes. **B**. The diagrams of zebrafish smyd1a genomic and protein structures. **C** and **D**. Sequence comparison of functional MYND (C) and SET (D) domains in Smyd1a and Smyd1b with other vertebrate Smyd1 and Smyd3 proteins. The identically conserved residues in the MYND and SET domains are indicated by the asterisk (*). The similar amino acid residues are indicated by = . ZF, zebrafish; Fu, fugu; Ch, chicken; Mo, mouse; Hu, human.
(PDF)

Figure S2 **Sequence alignment of vertebrate Smyd1 proteins.** Sequence comparison of zebrafish Smyd1a, Smyd1b, chicken Smyd1, mouse Smyd1 and human Smyd1 proteins.
(PDF)
