# Supplementary material for: Expression and Functional Characterization of Smyd1a in Myofibril Organization of Skeletal Muscles
Source: PLoS One. 2014 Jan 23;9(1):e86808. doi: 10.1371/journal.pone.0086808 (PMC3900645; doi:10.1371/journal.pone.0086808)
Supplement: Figure S1 — Structure comparison of zebrafish Smyd1a and Smyd1b. A. The synteny arrangement of zebrafish smyd1b and mouse smyd1 with fatty acid binding protein 1b (fabp1b) and thronine synthase like 2 (thnsl2) genes. B. The diagrams of zebrafish smyd1a genomic and protein structures. C and D. Sequence comparison of functional MYND (C) and SET (D) domains in Smyd1a and Smyd1b with other vertebrate Smyd1 and Smyd3 proteins. The identically conserved residues in the MYND and SET domains are indicated by the asterisk (*). The similar amino acid residues are indicated by = . ZF, zebrafish; Fu, fugu; Ch, chicken; Mo, mouse; Hu, human. (PDF) [file pone.0086808.s001.pdf]

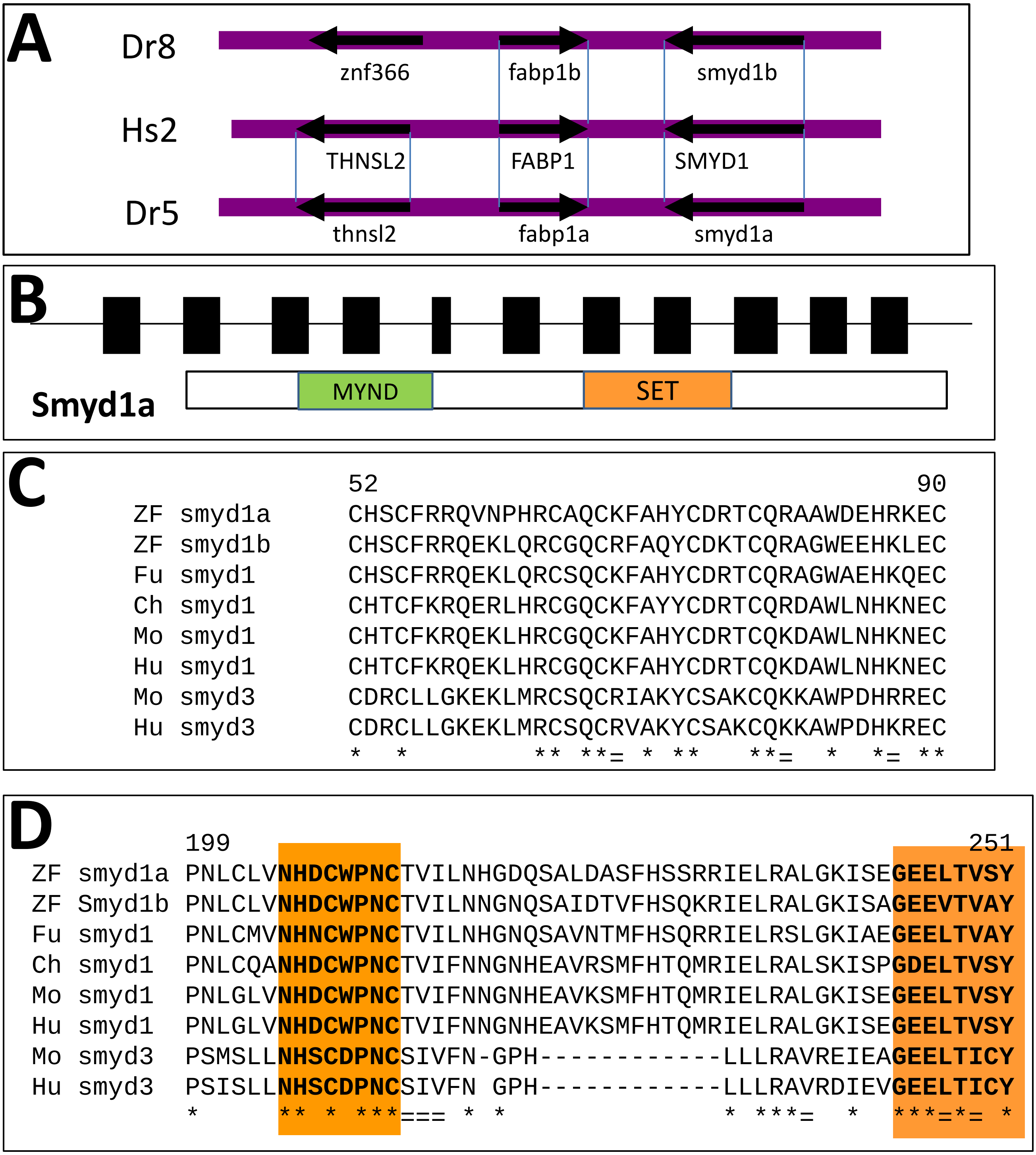

Figure S1. Structure comparison of zebrafish Smyd1a and Smyd1b

A. The synteny arrangement of zebrafish smyd1b and mouse smyd1 with fatty acid binding protein 1b (fabp1b) and thronine synthase like 2 (thnsl2) genes. **B.** The diagrams of zebrafish smyd1a genomic and protein structures. **C and D**. Sequence comparison of functional MYND (C) and SET (D) domains in Smyd1a and Smyd1b with other vertebrate Smyd1 and Smyd3 proteins. The identically conserved residues in the MYND and SET domains are indicated by the asterisk (*). The similar amino acid residues are indicated by =. ZF, zebrafish; Fu, fugu; Ch, chicken; Mo, mouse; Hu, human.
