# Supplementary material for: Expression and Functional Characterization of Smyd1a in Myofibril Organization of Skeletal Muscles
Source: PLoS One. 2014 Jan 23;9(1):e86808. doi: 10.1371/journal.pone.0086808 (PMC3900645; doi:10.1371/journal.pone.0086808)
Supplement: Figure S2 — Sequence alignment of vertebrate Smyd1 proteins. Sequence comparison of zebrafish Smyd1a, Smyd1b, chicken Smyd1, mouse Smyd1 and human Smyd1 proteins. (PDF) [file pone.0086808.s002.pdf]

Sequence alignment of vertebrate Smyd1 proteins

```
          1        10        20        30        40        50        60        70        80        90        100       110       120       130
          |--------+---------+---------+---------+---------+---------+---------+---------+---------+---------+---------+---------+---------|
 zfSmyd1a MTVEKTDPVEVFGAGLKGRGLRGTRDLSAGEVVFAEASFAAVVLDSLSLQVCHSCFRRQVNPHRCAQCKFAHYCDRTCQRAAWDEHRKECSAIRNIGKAPNENVRLVARILWRIQKHTGLVSDSQLTTLD
 zfSmyd1b      MEFVEVFDSPGKGRGLRATKEAWAGDVLFAEPPFASVVFDSQASSICHSCFRRQEKLQRCGQCRFAQYCDKTCQRAGWEEHKLECAAIKTYGKPPSENVRLAARILWRMDKQGSVVSDNQLTTLE
  chSmyd1      MESVEVFTTEGKGRGLKAQKEFLPGDVIFAEPAYAAVVFDSLTHVICHTCFKRQERLHRCGQCKFAYYCDRTCQRDAWLNHKNECSAIKKHGKAPTENIRLAARILWRIEREGGGLSENCLVSID
  moSmyd1 MTIGSMENVEVFTSEGKGRGLKATKEFWAADVIFAERAYSAVVFDSLINFVCHTCFKRQEKLHRCGQCKFAHYCDRTCQKDAWLNHKNECAAIKKYGKVPNENIRLAARIMWRVEREGTGLTEGCLVSVD
  HuSmyd1 MTIGRMENVEVFTAEGKGRGLKATKEFWAADIIFAERAYSAVVFDSLVNFVCHTCFKRQEKLHRCGQCKFAHYCDRTCQKDAWLNHKNECSAIKRYGKVPNENIRLAARIMWRVEREGTGLTEGCLVSVD
Consensus mt...m#.VEVF...gKGRGLratk#.wag#!.FAE..%aaVVfDSl...!CHsCFrRQeklhRCgQCkFAhYCDrTCQraaW.#Hk.ECsAIk.yGK.PnEN!RLaARI$WR.#k.g..vs#.qLttl#

          131      140       150       160       170       180       190       200       210       220       230       240       250       260
          |--------+---------+---------+---------+---------+---------+---------+---------+---------+---------+---------+---------+---------|
 zfSmyd1a MLEDHLSRMTPEDLKELKADVKTFYTYWPKKSKAVGEDYVSHLFGVISCNGFTLSDQRGLQSVGIGLFPNLCLVNHDCWPNCTVILNHGDQSALDASFHSSRRIELRALEPISAGQELTVSYVDFLSVST
 zfSmyd1b DLEDHICDISEDDLKDFKVDIHNFLDYWPRNSKPHTVDSVSHILGVINCNGFMVSDQRGLQAVGVGLFPNLCLVNHDCWPNCTVILNNGNQSAIDTVFHSQKRIELRALGKISAGEEVTVAYVDYLNVSA
  chSmyd1 DLQNHVESFDEEEKKDLRVDVESFLEFWPAQSQQFGMQYISHIFGVINCNAFTLSDQRGLQAVGVGIFPNLCQANHDCWPNCTVIFNNGNHEAVRSMFHTQMRIELRALSKISPGDELTVSYVDFLNVSE
  moSmyd1 DLQNHVEHFGEEEQKELRVDVDTFLQYWPPQSQQFSMQYISHIFGVINCNGFTLSDQRGLQAVGVGIFPNLGLVNHDCWPNCTVIFNNGNHEAVKSMFHTQMRIELRALGKISEGEELTVSYIDFLHLSE
  HuSmyd1 DLQNHVEHFGEEEQKDLRVDVDTFLQYWPPQSQQFSMQYISHIFGVINCNGFTLSDQRGLQAVGVGIFPNLGLVNHDCWPNCTVIFNNGNHEAVKSMFHTQMRIELRALGKISEGEELTVSYIDFLNVSE
Consensus dL##H.....e##lK#lkvD!.tFl.%WP..Sk....#y!SHifGVInCNgFtlSDQRGLQaVG!GlFPNLclvNHDCWPNCTVIlNnG#qsA.d..FHsq.RIELRALgkISaG#ElTVsY!D%LnvS.

          261      270       280       290       300       310       320       330       340       350       360       370       380       390
          |--------+---------+---------+---------+---------+---------+---------+---------+---------+---------+---------+---------+---------|
 zfSmyd1a DRQRLLQQQYYFDCKCEHCVNGTKDELMTAVKPTEDGKQPSADVVKQLTDFSLQALVKIEAARAQGNFHEVIRICRECLEKQDPVFADTNVHVLRVLSTASEVLSFLQQFQEAAGYAQRMVDGYMKLYHP
 zfSmyd1b DRQRLLKQQYFFDCTCKHCTEKIKDDLKMAGAEV-DGVKVPEEQVKEVTEFSRQKLEKMEKARIEANYNEVVKICRECVEKQENVLADTHIYYLRVCCTLSEVLSYLQFFDEASEYARKMVDGYLKLYHP
  chSmyd1 ERRKQLKKQYYFDCTCEHCKKKIKDDLMLAVKEG-DK-KPSAETVKEVIQFSKDTLEKINKARLEGTYHEVVKLCRECLKKQEPVLGDTNIYLLRILSIASEVLSYLQMFEEAAEYAKRMVEGYMKIYHP
  moSmyd1 ERRRQLKKQYYFDCSCEHCQKGLKDDLFLAAKE--DP-KPSQEVVKEMIQFSKDTLEKIDKARSEGLYHEVVKLCRECLEKQEPVFADTNLYVLRLLSIASEVLSYLQAYEEASHYARRMVDGYMKLYHH
  HuSmyd1 ERKRQLKKQYYFDCTCEHCQKKLKDDLFLGVKD--NP-KPSQEVVKEMIQFSKDTLEKIDKARSEGLYHEVVKLCRECLEKQEPVFADTNIYMLRMLSIVSEVLSYLQAFEEASFYARRMVDGYMKLYHP
Consensus #RqrlLkqQY%FDCtCeHC..k.KD#L..avke..#g.kps.#vVK#.t#FS.#.LeKi#kAR.#gn%hEV!kiCRECleKQ#pVfaDTniy.LRvlstaSEVLS%LQ.%#EAs.YArrMV#GY$KlYHp

          391      400       410       420       430       440       450       460       470       480       490493
          |--------+---------+---------+---------+---------+---------+---------+---------+---------+---------+--|
 zfSmyd1a NNAQLGMAIMRAGVTHWHAGLIEAAHGLICRAYGILMITHGPHHPISRDLEAMRRQTEMELRMFKQNEHVYHSMREAALTSKPMSVAGEQVQIKA
 zfSmyd1b NNAQLGMATMRAGVTHWQAGFIEVAHGMICKAFAILMITHGPTHPITKDLEAMRMQTEMELRMFQQNEYVYHSMREAALKNQPMRMMAEPTNEGIKNLFRRK
  chSmyd1 NNAQLGMAVMRAGVTHWHAGLIEAGHSMICKAYAILLITHGPSHPITKDLEVMRVQTEMELRMFQQNEFMYYKMREAALKNQKIQVMPEPSNEAPPSLFHKKE
  moSmyd1 NNAQLGMAVMRAGLTNWHAGHIEVGHGMICKAYAILLVTHGPSHPITKDLEAMRMQTEMELRMFRQNEFMYHKMREAALNNQPMQVMAEPSNEPAPALFHKKQ
  HuSmyd1 NNAQLGMAVMRAGLTNWHAGNIEVGHGMICKAYAILLVTHGPSHPITKDLEAMRVQTEMELRMFRQNEFMYYKMREAALNNQPMQVMAEPSNEPSPALFHKKQ
Consensus NNAQLGMA.MRAGvThWhAG.IEvaHg$ICkA%aIL$!THGP.HPItkDLEaMR.QTEMELRMF.QNE.vYhsMREAAL.nqpm.vmaEp.#e....lf..k.
```

Figure S2. Sequence alignment of vertebrate Smyd1 proteins. Sequence comparison of zebrafish Smyd1a, Smyd1b, chicken Smyd1, mouse Smyd1 and human Smyd1 proteins.
